# Rice receptor kinase FLR7 regulates rhizosphere oxygen levels and enriches the dominant *Anaeromyxobacter* that improves submergence tolerance in rice

**DOI:** 10.1093/ismejo/wrae006

**Published:** 2024-01-23

**Authors:** Hong-Bin Liu, Hong-Xia Sun, Li-Qiong Du, Ling-Li Jiang, Lin-An Zhang, Yin-Yao Qi, Jun Cai, Feng Yu

**Affiliations:** State Key Laboratory of Chemo/Biosensing and Chemometrics, College of Biology, Hunan University, Changsha 410082, P.R. China; Key Laboratory for Non-Wood Forest Cultivation and Conservation of Ministry of Education, College of Forestry, Central South University of Forestry and Technology, Changsha 410082, P.R. China; Interdisciplinary and Intelligent Seed Industry Equipment Research Department, Yuelushan Laboratory, Changsha 410082, P.R. China; State Key Laboratory of Chemo/Biosensing and Chemometrics, College of Biology, Hunan University, Changsha 410082, P.R. China; State Key Laboratory of Chemo/Biosensing and Chemometrics, College of Biology, Hunan University, Changsha 410082, P.R. China; State Key Laboratory of Chemo/Biosensing and Chemometrics, College of Biology, Hunan University, Changsha 410082, P.R. China; State Key Laboratory of Chemo/Biosensing and Chemometrics, College of Biology, Hunan University, Changsha 410082, P.R. China; State Key Laboratory of Chemo/Biosensing and Chemometrics, College of Biology, Hunan University, Changsha 410082, P.R. China; State Key Laboratory of Chemo/Biosensing and Chemometrics, College of Biology, Hunan University, Changsha 410082, P.R. China; State Key Laboratory of Chemo/Biosensing and Chemometrics, College of Biology, Hunan University, Changsha 410082, P.R. China; Interdisciplinary and Intelligent Seed Industry Equipment Research Department, Yuelushan Laboratory, Changsha 410082, P.R. China

**Keywords:** oxygen, the root microbiome, rice FLRs, Anaeromyxobacter, submergence tolerance

## Abstract

Oxygen is one of the determinants of root microbiome formation. However, whether plants regulate rhizosphere oxygen levels to affect microbiota composition and the underlying molecular mechanisms remain elusive. The receptor-like kinase (RLK) family member FERONIA modulates the growth–defense tradeoff in Arabidopsis. Here, we established that rice FERONIA-like RLK 7 (FLR7) controls rhizosphere oxygen levels by methylene blue staining, oxygen flux, and potential measurements. The formation of oxygen-transporting aerenchyma in roots is negatively regulated by FLR7. We further characterized the root microbiota of 11 FLR mutants including *flr7* and wild-type Nipponbare (Nip) grown in the field by 16S ribosomal RNA gene profiling and demonstrated that the 11 FLRs are involved in regulating rice root microbiome formation. The most abundant anaerobic-dependent genus *Anaeromyxobacter* in the Nip root microbiota was less abundant in the root microbiota of all these mutants, and this contributed the most to the community differences between most mutants and Nip. Metagenomic sequencing revealed that *flr7* increases aerobic respiration and decreases anaerobic respiration in the root microbiome. Finally, we showed that a representative *Anaeromyxobacter* strain improved submergence tolerance in rice via FLR7. Collectively, our findings indicate that FLR7 mediates changes in rhizosphere oxygen levels and enriches the beneficial dominant genus *Anaeromyxobacter* and may provide insights for developing plant flood prevention strategies via the use of environment-specific functional soil microorganisms.

## Introduction

Plant roots create environments for microbes in the rhizosphere (soil–root interface) and endosphere (internal tissues), forming taxonomically diverse microbial communities known as the root microbiome [1]. The root microbiota influences host plant fitness, including growth, nutrient uptake, pathogen resistance, and stress tolerance [2]. For example, the root microbiota has been found to help plants such as Arabidopsis (*Arabidopsis thaliana*), rice (*Oryza sativa* L.), and maize (*Zea mays* L.) absorb mineral elements such as nitrogen and iron [3–5]. Recent studies have supported that root microbial communities also enhance plant performance under abiotic stresses such as drought, salinity, and low light [6–8]. Given its importance in plant growth and health, the root microbiome has been referred to as the second genome of plants [9].

The root microbiome is shaped by complex interactions among the host, microbes, and the environment, and these interactions are controlled, at least in part, by plant genes [2]. Therefore, understanding the plant genetic control of the root microbiota is one of the prerequisites for the scientific and stable application of soil microbes for agricultural development. A growing number of studies have shown that root exudates and the plant immune system modulate root microbiome assembly [10]. Root-derived primary and secondary metabolites serve as nutrients and stimulators to drive microbial community assembly around roots [11–15]. Mutants with defects in Casparian strip synthesis and suberin deposition in the endodermis exhibit altered bacterial community composition, and interactions between the microbiota and root endodermis contribute to plant mineral nutrition homeostasis [16]. Moreover, microbiota composition is strongly correlated with environmental factors such as oxygen levels in soil [17]. Microbial tolerance to oxic settings is often used to classify microbes into obligate aerobes, facultative anaerobes, microaerophiles, and obligate anaerobes [18]. Soil oxygen levels fluctuate as a function of soil wetting and drying, with anoxic niches formed in the center of soil aggregates [19]. Although plant roots can alter oxygen availability in the rhizosphere by consuming or releasing oxygen and there exists a biologically relevant gradient of oxygen levels in soil [17, 20–22], whether plants regulate rhizosphere oxygen levels to further affect the microbiota and the underlying molecular mechanisms are unknown.

In addition to root cell respiration affecting rhizosphere oxygen content, in aquatic and wetland plants such as the important crop rice, oxygen is transported from the oxic shoots to anoxic roots via specialized aerenchyma and diffuses into the rhizosphere in a process known as radial oxygen loss (ROL) [23]. Aerenchyma size is proportional to root oxygen flux and may thus alter microbial oxygen availability and affect microbial community structure [23]. Studies have shown that aerenchyma formation is primarily stimulated by the plant hormone ethylene and that ethylene is regulated by complex genetic networks [24, 25]. Plasma membrane-localized receptor-like kinases (RLKs) constitute the largest receptor family in plants [26]. FERONIA (FER), a member of the RLK subfamily of *Catharanthus roseus* RLK1-like, negatively modulates ethylene biosynthesis in Arabidopsis, tomato (*Lycopersicon esculentum* Miller.), and apple (*Malus domestica*) [27, 28]. FER is also involved in the regulation of plant growth and immune responses to a variety of microbes [26, 29–32]. However, whether FER homologs mediate changes in rhizosphere oxygen levels and root microbiome formation in rice is unclear.

We have shown in previous studies that 16 FER-like RLKs (FLRs) in rice play different roles in the regulation of growth, grain size, and disease resistance [31, 33, 34]. In this study, we found that FLR7 controls rhizosphere oxygen levels and enriches the dominant genus *Anaeromyxobacter* in the root microbiota and that a representative *Anaeromyxobacter* strain contributes to submergence tolerance in rice via FLR7. Our study advances the understanding of the synergistic mechanisms underlying the interactions among plants, the environment, and microbes and provides clues for the engineering of hypoxia-dependent beneficial bacteria to address crop flooding.

## Results

### FERONIA-like RLK 7 negatively regulates rhizosphere oxygen levels

**Figure 1:**
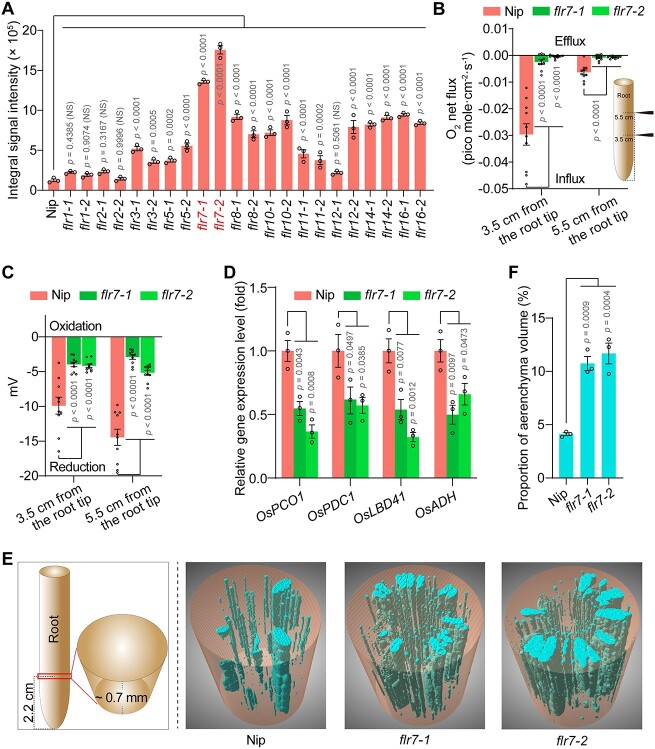
FLR7 negatively regulates rhizosphere oxygen levels; (A) quantified integral oxygen signal intensity within 1 cm from the root tip; (B) oxygen flux in the rhizosphere measured by NMT; positive values represent efflux from roots, and negative values represent influx into roots; a model diagram of the assay is presented; (C) potential in the rhizosphere measured by NMT; positive values represent oxidation potential, and negative values represent reduction potential; (D) relative expression levels of hypoxia-inducible marker genes in rice roots; (E) 3D-XRM of the aerenchyma near the root 2.2 cm away from the root tip; the distance between the root tip and the center of the examined ~0.7 mm thick root was 2.2 cm; a model diagram and representative 3D images are presented; (F) proportion of aerenchyma volume in E; in A–D and F, data are mean ± s.e.m. (*n* = 3 for each sample in A, D and F; *n* = 10 for each sample in B and C), and the significance of differences between the mutants and Nip was analyzed by ANOVA with Tukey’s HSD test; NS, not significant; *P* values are indicated; three independent experiments were performed with similar results.

We assessed ROL from rice roots of 11 FLR mutants (*flr1*, *flr2*, *flr3*, *flr5*, *flr7*, *flr8*, *flr10*, *flr11*, *flr12*, *flr14*, and *flr16*; information on the mutants, including agronomic traits such as growth, can be found in our previous studies [33, 34]; Table S1) and wild-type (WT) Nipponbare (Nip) by methylene blue staining. The results showed that the root tip color of the 11 mutants was darker than that of Nip to varying degrees, suggesting that these FLRs may control rhizosphere oxygen levels (Fig. S1A; Fig. 1A). Using the two lines of the most prominent mutant *flr7* as representatives, measurement of rhizosphere oxygen flux by noninvasive microtest technology (NMT) further showed that the rhizosphere oxygen levels of the mutant were significantly higher than those of Nip (Fig. 1B). Moreover, determination of the rhizosphere potential also confirmed that the rhizosphere of *flr7* was in an oxidative state relative to that of Nip (Fig. 1C). These results indicated that the oxygen content may have been higher in the mutant roots than in the Nip roots. We examined the expression levels of the hypoxia-inducible marker genes *OsPCO1* (*plant cysteine oxidase 1*), *OsPDC1* (*pyruvate dehydrogenase complex 1*), *OsLBD41* (*lateral organ boundaries domain 41*), and *OsADH* (*alcohol dehydrogenase*) in roots [35]. The results showed that the expression of these genes in the *flr7* roots was significantly downregulated compared with that in Nip roots, also suggesting that the mutant roots may have contained more oxygen (Fig. 1D). The aerenchyma is responsible for oxygen transport in rice roots [24]. We next analyzed whether the aerenchyma was altered in the mutant roots. The results obtained by 3D X-ray microscopy (3D-XRM) showed that aerenchyma formation was observably increased in the *flr7* roots compared with the Nip roots (Fig. 1E and F; Fig. S1B–D). These results reveal that FLR7 controls rhizosphere oxygen levels likely by negatively regulating aerenchyma formation.

### FER-like RLKs affect the composition of rice root microbiota, especially the dominant anaerobic-dependent genus *Anaeromyxobacter*

**Figure 2:**
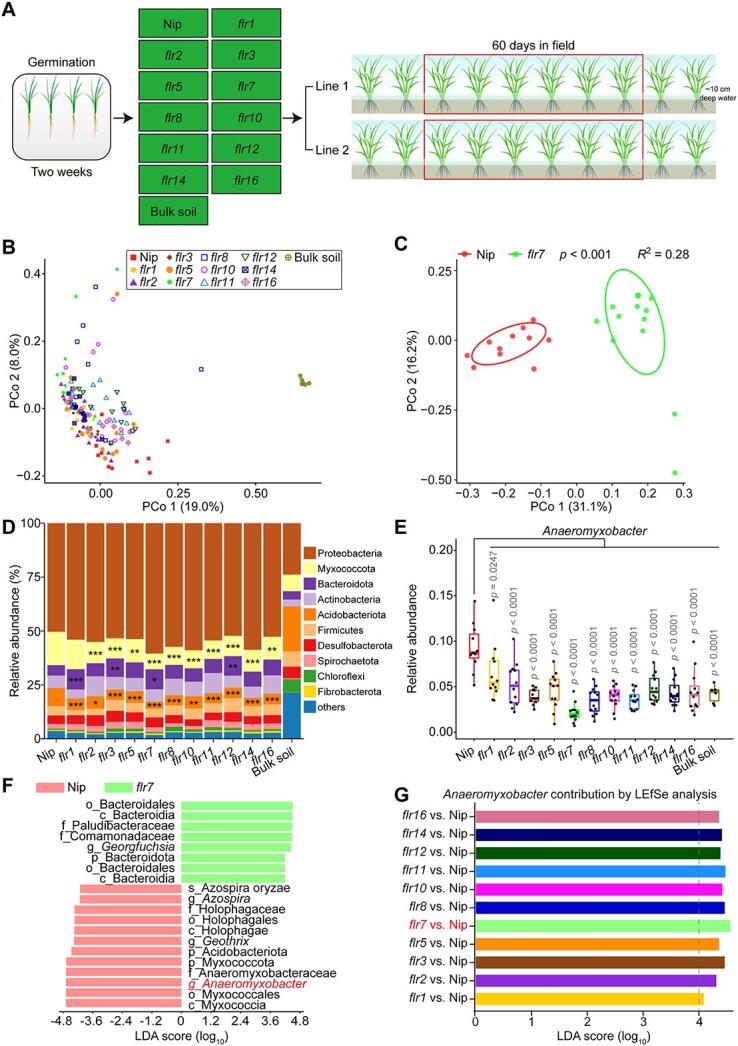
FLRs affect the composition of rice root microbiota, especially the dominant anaerobic-dependent genus *Anaeromyxobacter*;( A) diagram of the experimental design for rice field trials; (B) unconstrained PCoA with Bray–Curtis dissimilarity showing dissimilarity of bacterial diversity between 11 FLR mutants and Nip; (C) unconstrained PCoA with Bray–Curtis dissimilarity showing the separation of the root microbiota of *flr7* from that of Nip (*P* < .001, PERMANOVA by Adonis); ellipses cover 68% of the data for each rice genotype; (D) phylum-level distribution of the root microbiota of Nip, 11 FLR mutants, and bulk soil; the significance of differences in the relative abundance of each phylum between the mutants and Nip was analyzed by ANOVA with Tukey’s HSD test; ^*^^*^^*^P < .001; ^*^^*^P < .01; ^*^P < .05; exact *P* values are provided in Table S6; (E) relative abundance of the most dominant genus *Anaeromyxobacter* in Nip, 11 FLR mutants, and bulk soil; box plots show the median with upper and lower quartiles, and whiskers present the 1.5× interquartile range; statistical significance was determined by ANOVA with Tukey’s HSD test and *P* values are indicated; (F) LEfSe analysis of the root microbiota between Nip and *flr7*; the LDA score represents the contribution of differential lineages; lineages with LDA > 4 are displayed, and p, c, o, f, g, and s are the bacterial taxon abbreviations for phylum, class, order, family, genus, and species, respectively; (G) LEfSe analysis of the contribution of *Anaeromyxobacter* to differences in the root microbiota between Nip and each FLR mutant; the numbers of replicated samples in this figure are as follows: Nip (*n* = 11), each FLR mutant (*n* = 12), and bulk soil (*n* = 6).

To investigate whether FLR-mediated changes in rhizosphere oxygen levels affect the assembly of the rice root microbiota, we grew two independent lines of the 11 FLR mutants and WT Nip in an experimental field that had been used to grow rice for the past 10 years in Changsha, Hunan Province, China. This enabled us to examine stable patterns of root microbiota membership between different genotypes of rice in representative agricultural soil. To avoid disturbance from seed-associated microorganisms, we germinated dehulled and sterilized rice seeds on Murashige and Skoog (MS) agar medium and transferred 2-week-old seedlings to the field. The root-associated microbiota was sampled 60 days after the rice seedlings were transplanted (the heading stage), when the root microbiota was well established and stable [36, 37]. For each mutant, we harvested 12 root samples from six representative plants of Line 1 and six representative plants of Line 2, and we also collected 11 root samples from 11 representative Nip plants and six unplanted soil samples from the same field (Fig. 2A). We generated a bacterial community profile for each sample via polymerase chain reaction (PCR) amplification of the 16S ribosomal RNA (rRNA) gene targeting regions V5–V7 using the primers 799F and 1193R (Table S2), followed by NovaSeq 6000 sequencing. We obtained 10 487 785 high-quality sequences from 150 samples (average of 70 388 reads and range of 51 235–91 299 reads per sample). We analyzed high-quality reads with USEARCH, removing chimeric and organelle sequences, to produce 322 584 operational taxonomic units (OTUs; mean, 2165 OTUs per sample). Rarefaction and species accumulation analysis revealed that our population captured most root microbiota members from each rice genotype, and our sample number was sufficient (Fig. S2A and B).

Unconstrained principal coordinate analysis (PCoA) of Bray–Curtis dissimilarity and permutational multivariate analysis of variance (PERMANOVA) revealed that the composition of the root bacterial microbiota differed between Nip and the 11 FLR mutants (Fig. 2B and C; Fig. S3). Of these, the community structure of *flr7* changed the most according to the two larger separations shown in the PCoA results (Fig. 2C; Fig. S3). Measurement of within-sample diversity (α-diversity) showed no significant differences in bacterial diversity between Nip and the 11 mutants (Fig. S2C), while the differences in the root microbiota between Nip and the 11 mutants were detectable and significant at the phylum level (Fig. 2D). Myxococcota was regulated by most FLRs, and its relative abundance was significantly reduced in all mutants except *flr1* compared to Nip. These data indicate that these 11 FLRs play important roles in rice root microbiome formation.

We observed changes in the abundance of numerous bacterial genera within the root mycobiota of these mutants compared to that in the Nip root mycobiota (Fig. S4; Table S3). The relative abundance of the anaerobic-dependent genus *Anaeromyxobacter* (belonging to Myxococcota [38]), with the highest relative abundance in the Nip root microbiota, was significantly reduced in all the mutants compared to Nip (Fig. 2E). Since *flr7* was the mutant with the most changes in the root microbiota compared to Nip, we further analyzed the differences between this mutant and Nip at the OTU level. Venn diagrams, Manhattan plots, and the bacterial co-occurrence network showed that the root microbiota underwent dramatic changes in *flr7* compared to Nip (Fig. S5A–C). To identify the bacterial genus with the greatest influence on the difference in the microbiota between the mutant and Nip, we performed LDA effect size (LEfSe) analysis and found that the deletion in *flr7* of the dominant genus *Anaeromyxobacter* contributed the most to the changes in the root microbiota (Fig. 2F). Similarity percentage (Simper) analysis of species contribution also yielded results consistent with this observation (Fig. S5D). We also observed that *Anaeromyxobacter* was the common biomarker for differences in the root microbiota between each FLR mutant and Nip (Fig. 2G). These results suggest that FLRs are responsible for the assembly of the root-dominant genus *Anaeromyxobacter*.

### FERONIA-like RLK 7 mutation enhances aerobic characteristics and diminishes anaerobic characteristics in the root microbiome

**Figure 3:**
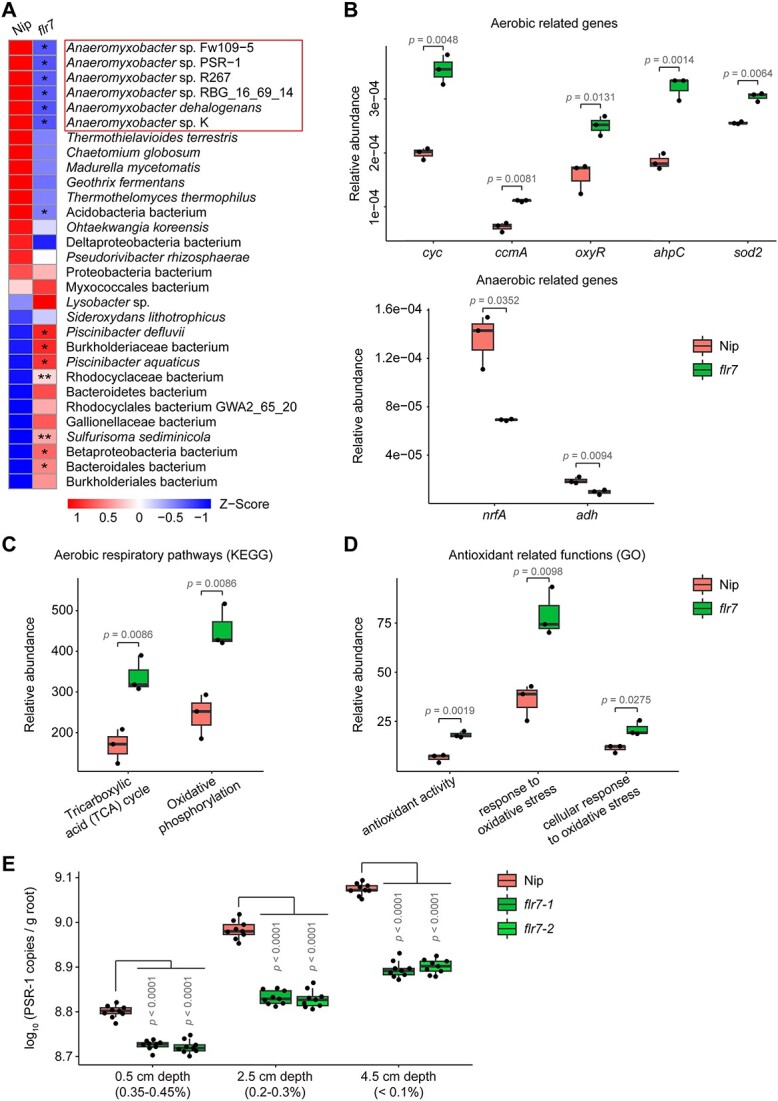
FLR7 mutation enhances aerobic characteristics and diminishes anaerobic characteristics in the root microbiome; (A) differences in the relative abundance of species between the Nip and *flr7* root metagenomes; the top 30 species in relative abundance in the Nip root metagenome are presented; relative abundance of aerobic and anaerobic respiration-related genes (B), aerobic respiratory pathways (C) and antioxidant-related functions (D); (E) bacterial copies of *Anaeromyxobacter* sp. PSR1 in the rice rhizosphere after 30 days of culture at different water depths (oxygen content); in A–D, the significance of differences in the relative abundance of each species or gene or pathway and function between *flr7* and nip was analyzed by Welch’s *t*-test, and the numbers of replicated samples are as follows: Nip (*n* = 3) and *flr7* (*n* = 3). In a, ^*^^*^P < .01; ^*^P < .05; exact *P* values are provided in Table S6; in B–D, *P* values are indicated; in E, the significance of differences in the number of bacterial copies between the mutants and Nip was analyzed by ANOVA with Tukey’s HSD test, and the numbers of replicated samples are as follows: Nip (*n* = 9), *flr7–1* (*n* = 9), and *flr7–2* (*n* = 9); *P* values are indicated; box plots show the median with upper and lower quartiles, and whiskers present the 1.5× interquartile range.

To address the role of FLR7 in the root microbiome, we carried out metagenomic sequencing of the *flr7* root microbiome. After removing plant-derived sequences, we obtained an average of 9.28 gigabytes of microbial sequences per sample. We used a de novo assembly of reads to predict and annotate genes against the nonredundant protein sequence database and Kyoto Encyclopedia of Genes and Genomes (KEGG) or Gene Ontology (GO) databases for species and gene function analysis, respectively. Gene rarefaction analysis revealed that the sample sequencing depth was adequate for subsequent analysis (Fig. S6A). Unconstrained PCoA results indicated that the species composition in the root microbiome of *flr7* differed from that of Nip (Fig. S6B). Analysis of the top 30 species in terms of relative abundance showed that multiple species in the dominant genus *Anaeromyxobacter*, such as *Anaeromyxobacter* sp. Fw109-5, *Anaeromyxobacter* sp. PSR-1, and *Anaeromyxobacter* sp. R267, were all significantly reduced in the *flr7* root microbiome compared to the Nip root microbiome (Fig. 3A).

Since FLR7 affects rhizosphere oxygen levels, we further analyzed genes related to aerobic and anaerobic respiration. The results indicated that the relative abundances of five representative aerobic respiration-related genes, *cyc* (expressing cytochrome c), *ccmA* (expressing heme exporter protein A), *oxyR* (expressing hydrogen peroxide-inducible gene activator), *ahpC* (expressing peroxiredoxin), and *sod2* (expressing superoxide dismutase 2) [39], were significantly increased, while the relative abundances of two representative anaerobic respiration-related genes, *nrfA* (expressing nitrite reductase) and *adh* (expressing alcohol dehydrogenase) [40], were significantly decreased in the *flr7* root microbiome compared to the Nip root microbiome (Fig. 3B; Table S4). KEGG pathway analysis revealed that the relative abundances of the key pathways of the tricarboxylic acid cycle and oxidative phosphorylation during aerobic respiration were greatly increased in the mutant root microbiome (Fig. 3C). Aerobic respiration is accompanied by antioxidant reactions that exist in aerobic bacteria but not in anaerobic bacteria [39]. GO function analysis showed that the relative abundances of multiple antioxidant-related functions were significantly elevated in the mutant root microbiome (Fig. 3D). These results suggest that rhizosphere oxygen levels controlled by FLR7 mediate changes in the abundance of aerobic and anaerobic bacteria.

We used the strain PSR-1 isolated from paddy soil as the representative *Anaeromyxobacter* [41] to establish that the abundance of *Anaeromyxobacter* in the *flr7* rhizosphere was significantly reduced relative to that in the Nip rhizosphere, as determined by single inoculation experiments (Fig. 3E). Due to the infeasibility of directly removing *flr7* rhizosphere oxygen, we set different water depths in the culture system to generate different rhizosphere oxygen levels without threatening the health of rice. The results of differential PSR-1 colonization in the *flr7* rhizosphere at different water depths further verified that FLR7-regulated rhizosphere oxygen levels were the direct cause of the changes in *Anaeromyxobacter* abundance (Fig. 3E). Our data indicate that FLR7 controls rhizosphere oxygen levels and thus enriches dominant anaerobic-dependent *Anaeromyxobacter* in the rice root microbiome.

### *Anaeromyxobacter* improves submergence resistance in rice dependent on FLR7 via a low-oxygen quiescence-based strategy

**Figure 4:**
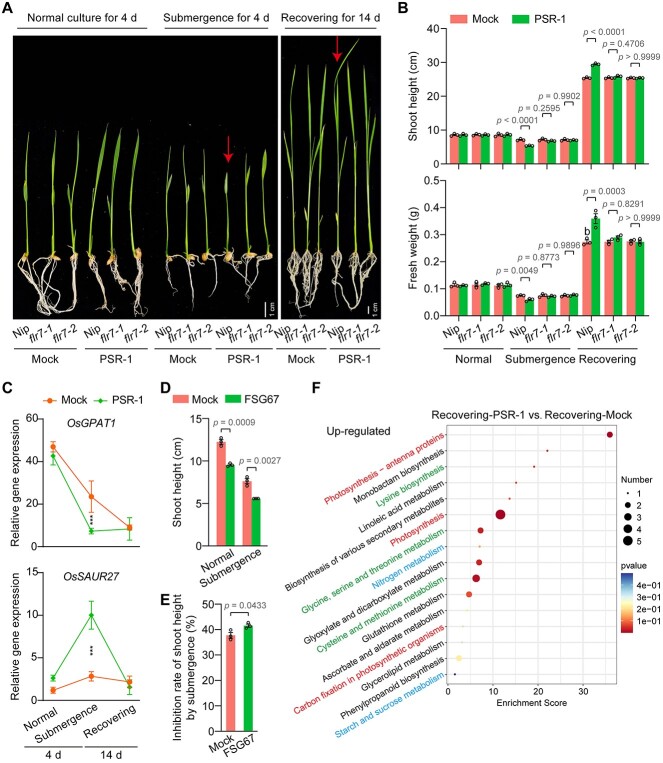
*Anaeromyxobacter* improves submergence resistance in rice dependent on FLR7 via a low-oxygen quiescence-based strategy; (A) representative images of rice on the indicated days in normal culture, submergence treatment or recovery treatment after root inoculation with the *Anaeromyxobacter* strain PSR-1; (B) shoot height and fresh weight of rice for each treatment in A; (C) expression levels of the positive growth regulator *OsGPAT1* and the negative growth regulator *OsSAUR27* in the transcriptomes of each treatment; *P* < .05 and fold change >1.5 or < 0.5 (DESeq2) were set as the thresholds for significant differential expression; ^*^^*^^*^P < .001; (D) effects of 50 μM GPAT inhibitor FSG67 on rice shoot height under normal culture and submergence stress; DMSO (0.5%; v/v; mock) was used as a negative control; (E) rate of inhibition of shoot height by submergence under treatment with the GPAT inhibitor FSG67; (F) top 20 pathways from KEGG enrichment analysis based on the *P* values for genes that showed upregulation with recovering-PSR-1 treatment compared with recovering-mock treatment; in B–E, the data are presented as the mean ± s.e.m. of three biological replicates; in B, data were analysed by ANOVA with Tukey’s HSD test and partial *P* values are indicated; *P* values for additional comparisons are provided in Table S6; in D and E, the significance of differences between GPAT inhibitor treatment and mock treatment was analyzed by Student’s *t*-test and *P* values are indicated; three independent experiments were performed with similar results.

It is unknown how the root-dominant genus *Anaeromyxobacter* affects rice. Given that *Anaeromyxobacter* survives in anaerobic environments, we explored the effect of a representative *Anaeromyxobacter* strain, PSR-1, on rice during submergence stress. Submergence treatment was performed after inoculating the roots of 4-day-old sterile Nip and *flr7* seedlings with PSR-1 (Fig. S7A). We found that PSR-1 observably slowed the growth of Nip submerged for 4 days compared to the uninoculated mock treatment but did not affect the growth of Nip normally cultured for 4 days (Fig. 4A and B; Fig. S7B). We then returned the submergence treatment to normal culture and surprisingly found that the Nip seedlings inoculated with PSR-1 grew significantly stronger than the uninoculated mock seedlings after recovery for 14 days (Fig. 4A and B; Fig. S7B). The slowed growth during submergence and the accelerated growth after recovery conferred by PSR-1 were almost abolished in *flr7* seedlings (Fig. 4A and B; Fig. S7B). We also found that a facultative anaerobic control bacterium, *Escherichia coli*, did not affect the growth of Nip seedlings submerged for 4 days compared to the uninoculated mock seedlings (Fig. S7C and D). These results indicate that PSR-1 improves submergence resistance in rice dependent on FLR7 through a low-oxygen quiescence-based strategy by which shoots do not elongate upon submergence to retain energy for later growth [42].

To understand the molecular mechanism by which PSR-1 acts on rice, we performed transcriptome sequencing analysis of Nip. Transcriptome data were validated reliably by detecting changes in the expression of 10 significant differentially expressed genes via quantitative real-time PCR (qRT–PCR) (Fig. S8A). The KEGG enrichment analysis also showed that multiple photosynthesis-related pathways and metabolic processes were downregulated in rice under submergence treatment compared to normal culture (Fig. S8B), consistent with previous studies [43–45]. We found that PSR-1 significantly decreased the expression levels of the positive growth regulator *OsGPAT1* [46] and significantly increased the expression levels of the negative growth regulator *OsSAUR27* [47] compared to the uninoculated mock and *E. coli* treatment when rice was submerged for 4 days (Fig. 4C; Fig. S8C). Moreover, the expression of these two genes did not show significant differences between the PSR-1 treatment and uninoculated mock treatment at 4 days of normal culture and 14 days of recovery (Fig. 4C; Table S5). We further confirmed the importance of *OsGPAT1* for rice growth under normal culture conditions and submergence stress using the GPAT inhibitor FSG67 (Fig. 4D and E; Fig. S8D and E). After recovery for 14 days, we found that KEGG processes closely related to growth, such as photosynthesis-related pathways, amino acid metabolism, nitrogen metabolism, and starch and sucrose metabolism, were upregulated in rice inoculated with PSR-1 (Fig. 4F). These results indicate that PSR-1 functions via a low-oxygen quiescence-based strategy under submergence stress, likely by manipulating the expression of rice growth-related genes.

## Discussion

Root microbiota assembly is crucial for plant fitness, and developing genetic regulation of root microbiome formation is necessary for the rational application of beneficial microbes in crop production [2]. Herein, our results show that the receptor kinase FLR7 mediates changes in rhizosphere oxygen levels and regulates rice root microbiota composition, especially the abundance of the beneficial dominant genus *Anaeromyxobacter*. The three factors that determine the composition and function of the root microbiome—plant, microbes, and the soil environment—have rarely been integrated to elucidate the mechanisms underlying root microbiome formation [2]. This study links plant genetic molecules, the environmental factor oxygen, and root microbiota composition, although it was found that roots can influence the rhizosphere by altering soil oxygen availability, pH, etc. [20] and that different microbes adapt differently to environmental factors such as oxygen and pH [17]. Gaining further insight into the molecular mechanisms of plant–soil–microbe interactions is essential for obtaining a comprehensive understanding of root microbiome formation and optimal utilization of the root microbiota.

We dissected into how FLR7 controls rhizosphere oxygen levels and found a likely key factor, the oxygen-transporting aerenchyma, whose formation is negatively regulated by FLR7. Unfortunately, the underlying molecular pathways by which FLR7 is involved in aerenchyma formation are unknown and require further exploration. The anaerobic-dependent genus *Anaeromyxobacter* is a dominant genus in most of the reported rice root microbiomes, including in this study [48, 49]. We found that the receptor kinase FLR7 determines the enrichment of *Anaeromyxobacter* in rice root microbiota by reducing rhizosphere oxygen levels. Although oxygen levels near rice roots are generally relatively higher than those in unplanted soil due to root oxygen secretion and tight differences [17, 50], the abundance of *Anaeromyxobacter* in rice root microbiota is significantly higher than that in unplanted soil. This suggests that rice roots can also enrich *Anaeromyxobacter* in a specific manner within certain oxygen concentrations, probably due to nutrients secreted by the roots [21]. We do not rule out the existence of other genes that may also control rhizosphere oxygen levels to influence *Anaeromyxobacter* assembly, given the potential factors affecting oxygen, such as the ROL barrier, respiration, and photosynthesis [24]. Whether FLR7 enriches *Anaeromyxobacter* through other pathways, such as primary and secondary metabolites, also needs further investigation. Mutations in all 11 tested FLRs significantly reduced the abundance of *Anaeromyxobacter* in the root microbiome, consistent with the increased rhizosphere oxygen levels of these mutants, although different FLRs differentially altered the abundance of many other genera. The effects of FLRs on microbes other than *Anaeromyxobacter* will be a focus of future work, and whether the finding that FLR7 mediates rhizosphere oxygen changes and affects root microbiota is generalized in other aquatic plants with aerenchyma remains open to exploration.

What is the role of rice roots enriching large amounts of *Anaeromyxobacter* from bulk soil? The root microbiota influences plant nutrient uptake and resistance to biotic and abiotic stresses [10]. The severe flooding caused by climate change and the waterlogged environment in which rice is cultivated inspired us to investigate the effect of *Anaeromyxobacter* on rice submergence resistance. Surprisingly, our results showed that an *Anaeromyxobacter* strain enhanced submergence resistance in rice by modulating the expression of rice growth-related genes, although whether other root-associated microbes can play a similar role remains unclear. A previous study confirmed that multiple *Anaeromyxobacter* strains contained the minimum set of genes for nitrogenase activity (*nifBHDKEN*) and could fix nitrogen in the paddy soil environment [41]. This suggests that *Anaeromyxobacter* may also help rice absorb mineral nitrogen. Both *Anaeromyxobacter* and rice thrive in waterlogged soil environments, and long-term coexistence may have led to their coevolution and mutually beneficial relationship [51]. Further elucidation of the molecular mechanism underlying the interaction between *Anaeromyxobacter* and rice is important for the expansion of theoretical knowledge and the utilization of microbial resources.

In summary, our data indicate that the rice receptor kinase FLR7 plays a crucial role in maintaining root microbiota homeostasis and highlight a connection among the host, environment, and microbes.

## Materials and methods

### Plant materials and culture

WT Nip and 11 CRISPR–Cas9 mutants (*flr1*, *flr2*, *flr3*, *flr5*, *flr7*, *flr8*, *flr10*, *flr11*, *flr12*, *flr14*, and *flr16*) were used in this study, and the agronomic traits of these mutants, such as growth, have been reported in our previous studies [33, 34]. Rice seeds were dehulled, surface-sterilized in 75% ethanol for 30 s, and treated with 2.5% sodium hypochlorite three times for 15 min each. Subsequently, the seeds were germinated on MS agar medium. Then, seedlings were transferred to Kimura B nutrient solution (pH 5.8) or soil or sand to be cultured under long-day conditions (16 h light/8 h dark cycles) at 26°C.

### Methylene blue staining of radial oxygen loss from rice roots

The redox indicator dye methylene blue was used to qualitatively assess ROL from 11 mutants and WT Nip roots. Methylene blue (13 mg/L) was mixed with 0.1% (w/v) agar and 130 mg/L sodium dithionite (Na_2_S_2_O_4_) to generate a deoxidized colorless test solution [52]. One adventitious root that was ~6 cm long was picked from each hydroponic 20-day-old plant, and the other roots were trimmed off. The adventitious roots from the mutants and Nip were then transferred to the test solution in an acrylic tank and held with the root–shoot junction positioned 3 cm below the surface of the solution. After 20 min, root staining patterns within 1 cm from the root tip were photographed and quantified using ImageJ software after the background was subtracted and the area was unified. Each genotype contained six replicates.

### Measurement of oxygen flux and potential in the rhizosphere

We used *flr7* as a representative for further detection. The rhizosphere oxygen flux and potential of adventitious roots that were ~8 cm in length of the mutant and Nip grown in soil for 20 days were measured by NMT (Xuyue Sci. & Tech. Co., Ltd, Beijing, China). Each genotype contained 10 replicates. After immobilizing the roots on the bottom of the Petri dish using filter paper and resin, the roots were immersed for 20 min with nitrogen-filled hypoxic test solution (0.1 mM CaCl_2_, pH = 6.0), and nitrogen was continuously pumped into the solution. Sites at 3.5 cm and 5.5 cm from the root tip were selected as test sites. After the hypoxic test solution was replaced with fresh solution, an oxygen flux microsensor was placed at a distance of ~30 μm from the surface of the test site under the microscope for detection for 5 min. A membrane potential microelectrode was placed at a distance of 2–3 μm from the surface of the test site to measure the stable potential. The software imFluxes V.3.0 (YoungerUSA LLC, Amherst, MA 01002, USA) was used to obtain oxygen flux or potential data, with positive values representing efflux from the root or oxidation potential and negative values representing influx into the root or reduction potential.

### Detection of aerenchyma formation in roots

Adventitious roots ~8 cm long with uniform growth of hydroponic 20-day-old rice seedlings were selected, and root segments from 2.0 to 2.4 cm from the root tip were used for 3D-XRM by ZEISS Xradia 515 Versa (Carl Zeiss AG, Germany). A local magnification of ~0.7 μm × 0.7 μm × 0.7 μm voxels was performed by centering at a site 2.2 cm or 2.0 cm from the root tip for aerenchyma imaging. A total of 1001 images were acquired with 60 V voltage and 5000–6000 intensity. Dragonfly V0.6.0 was used for image processing and quantitative analysis of aerenchyma volume.

### Plant materials, culture, and soil property measurements for characterizing root microbiota

Rice seeds from the same batch of Nip and two independent lines of 11 FLR mutants (*flr1*, *flr2*, *flr3*, *flr5*, *flr7*, *flr8*, *flr10*, *flr11*, *flr12*, *flr14*, and *flr16*) were dehulled, surface-sterilized, and germinated on MS agar medium. Then, 2-week-old rice seedlings were transplanted into fields on Yujialaowu Farm, Hunan Province, China (28.573° N, 113.339° E), on April 29, 2021; these fields have been used to grow rice for the past 10 years. Ten individuals of each rice genotype were planted in a row with a spacing of 20 cm, and the planting distance between different genotypes was set to 60 cm for protection (Fig. 2A). The physicochemical properties of three soil samples from depths of 5–6 cm in unplanted areas were measured by Shanghai Weike Testing Co., Ltd (Shanghai, China) and Hunan Institute of Microbiology (Changsha, China) according to the standard methods. The soil analysis data were as follows: pH 6.4 ± 0.14; organic matter levels of 10.9 ± 0.63 g/kg; available N, P, and K levels of 59.47 ± 2.66, 12.57 ± 0.76, and 167.25 ± 18.36 mg/kg, respectively; and micronutrient Fe, Mn, Cu, and Zn levels of 38767.5 ± 4482.63, 776.3 ± 102.8, 17.6 ± 0.73, and 65.4 ± 0.9 mg/kg, respectively.

### Sample collection

Sixty days after the seedlings were transplanted, root samples from rice plants in the late tiller stage were collected at a depth of 0–10 cm. Six representative individuals from each genotype were selected from a central position. Roots were shaken to remove loosely attached soil and washed in 25 ml of sterile phosphate-buffered saline in a 50 ml Falcon tube until no visible soil particles remained. After being dried on sterile filter paper, the roots were cut into 2 mm sections and placed into 2 ml tubes [3]. In total, 143 rice root samples and six unplanted bulk soil samples were collected. All samples were immediately placed on dry ice, transported to the laboratory, and stored at −80°C.

### DNA extraction, PCR amplification, and sequencing

DNA from frozen root and bulk soil samples was extracted with the FastDNA SPIN Kit (MP Biomedicals) according to the manufacturer’s instructions. The DNA concentration was determined using the PicoGreen dsDNA Assay Kit (Life Technologies, USA) and diluted to 3.5 ng μl^−1^. The variable regions V5–V7 of the bacterial 16S rRNA gene were amplified with the primers 799F and 1193R [53]. Each sample was amplified in triplicate in a 30 μl reaction system containing 3 μl of DNA, 0.75 U of PrimeSTAR HS DNA Polymerase, 1× PrimeSTAR Buffer (TaKaRa, Japan), 0.2 mM deoxyribonucleoside triphosphates, and 10 pM barcoded forward and reverse primers with linker regions. The three PCR products were purified together using an AMPure XP Kit (Beckman Coulter, USA), measured with a Nanodrop (NanoDrop 2000C, Thermo Scientific), and diluted to 10 ng μl^−1^ as templates for the second-step PCR using Illumina-compatible primers. All samples were amplified in triplicate for eight cycles, and triplicate PCR products from each sample were pooled and run on a 1.2% (w/v) agarose gel. The amplicons were extracted by a QIAquick Gel Extraction Kit (Qiagen, USA) and measured using a PicoGreen dsDNA Assay Kit. Two hundred nanograms of each sample were mixed, and the final amplicon libraries were purified twice with an AMPure XP Kit and subjected to a single sequencing run on the NovaSeq 6000 platform (Illumina Inc., USA).

### Bioinformatics analysis of 16S rRNA gene profiling

The quality of the paired-end Illumina reads was checked by FastQC v.0.11.6, and then these reads were assigned to samples according to the unique barcode. After barcodes and primers were removed, the paired-end reads were merged using FLASH v.1.2.7, and low-quality reads were filtered with QIIME v.1.9.1. With the sequences from chimera and host plastids being further removed based on the SILVA 138 database by USEARCH v.10.0, unique reads were clustered into OTUs with 97% similarity. Representative sequences for each OTU were picked by UPARSE v.7.0.1001, and taxonomic information was classified with the RDP classifier. Twelve samples from two independent lines of each mutant were combined for subsequent analysis. Rarefaction curve analysis was performed with the vegan (rrarefy function; 23 498 reads) and ggplot2 packages in R v.4.1.2. Diversity analysis was performed using QIIME2 and R. Analysis of the differential OTU abundance and taxa was carried out by Wilcoxon rank sum tests based on OTUs with median relative abundance from each genotype >0.2%, and corresponding *P* values were corrected for multiple tests with the false discovery rate (FDR) set at 0.05. Unconstrained PCoA of Bray–Curtis dissimilarity and PERMANOVA were performed using the vegan, ape, and ggplot2 packages in R. Contribution analysis of LEfSe and Simper was carried out with LEfSe software and the vegan package in R, respectively. The cooccurrence network was analyzed with the psych package in R and Gephi v.0.9.2.

### Supporting information for materials and methods

Details are supplied in the Supporting Information for the experimental programs for analysis of the expression of hypoxia-inducible marker genes in roots, metagenomic analysis, determination of *Anaeromyxobacter* abundance in the rice rhizosphere, root inoculation with *Anaeromyxobacter* and submergence assay, transcriptome sequencing analysis, and analysis of the effect of a GPAT inhibitor on rice growth.

### Statistical analysis

Three biological replicates with at least three technical replicates were used for measurements of ROL and oxygen flux and potential in the rhizosphere, qRT–PCR, determination of aerenchyma and *Anaeromyxobacter* abundance, and submergence assay. The mean and standard error of the mean (s.e.m.) values of the biological replicates were calculated using Microsoft Excel software (Microsoft Corp., Redmond, WA). One-way analysis of variance (ANOVA) (Tukey’s HSD test) and Student’s or Welch’s *t*-test were conducted using GraphPad software or SPSS software (ver. 23.0; IBM China Company Ltd, Beijing, China) to determine the statistical significance. *P* < .05 was considered to indicate a significant difference, and exact *P* values were rounded to four decimal places. Detailed data analysis information is provided in Table S6.

## Supplementary Material

Table_S1_Supplementary_mutation_information_for_FLRs_wrae006

Table_S2_Specific_primers_used_in_this_study_wrae006

Table_S3_Relative_abundance_of_bacterial_genera_wrae006

Table_S4_Relative_abundance_of_genes_wrae006

Table_S5_Significant_differentially_expressed_genes_wrae006

Table_S6_Data_analysis_wrae006

Supplementary_figures_and_table_legends_wrae006

Supporting_information_for_materials_and_methods_wrae006

## Data Availability

The data that support the findings of this study are available from the corresponding author upon reasonable request. Raw sequence data reported in this paper have been deposited (PRJCA014387) in the Genome Sequence Archive in the BIG Data Center (http://bigd.big.ac.cn/gsa) [54], Chinese Academy of Sciences under accession codes CRA009552 for bacterial 16S rRNA gene sequencing data, CRA009562 for metagenomic sequencing data, and CRA009561 for transcriptome sequencing data.
