# Supplementary material for: Rice receptor kinase FLR7 regulates rhizosphere oxygen levels and enriches the dominant Anaeromyxobacter that improves submergence tolerance in rice
Source: ISME J. 2024 Jan 23;18(1):wrae006. doi: 10.1093/ismejo/wrae006 (PMC10900889; doi:10.1093/ismejo/wrae006)
Supplement: Supplementary_figures_and_table_legends_wrae006 [file supplementary_figures_and_table_legends_wrae006.docx]

**Supplementary table legends**

**Table S1.** Supplementary mutation information for FLRs

**Table S2.** Specific primers used in this study

**Table S3.** Relative abundance of bacterial genera in the 16S rRNA sequencing data

**Table S4.** Relative abundance of genes in the metagenomes

**Table S5.** Significantly differentially expressed genes in the transcriptomes

**Table S6.** Data analysis

**Supplementary figures and legends**


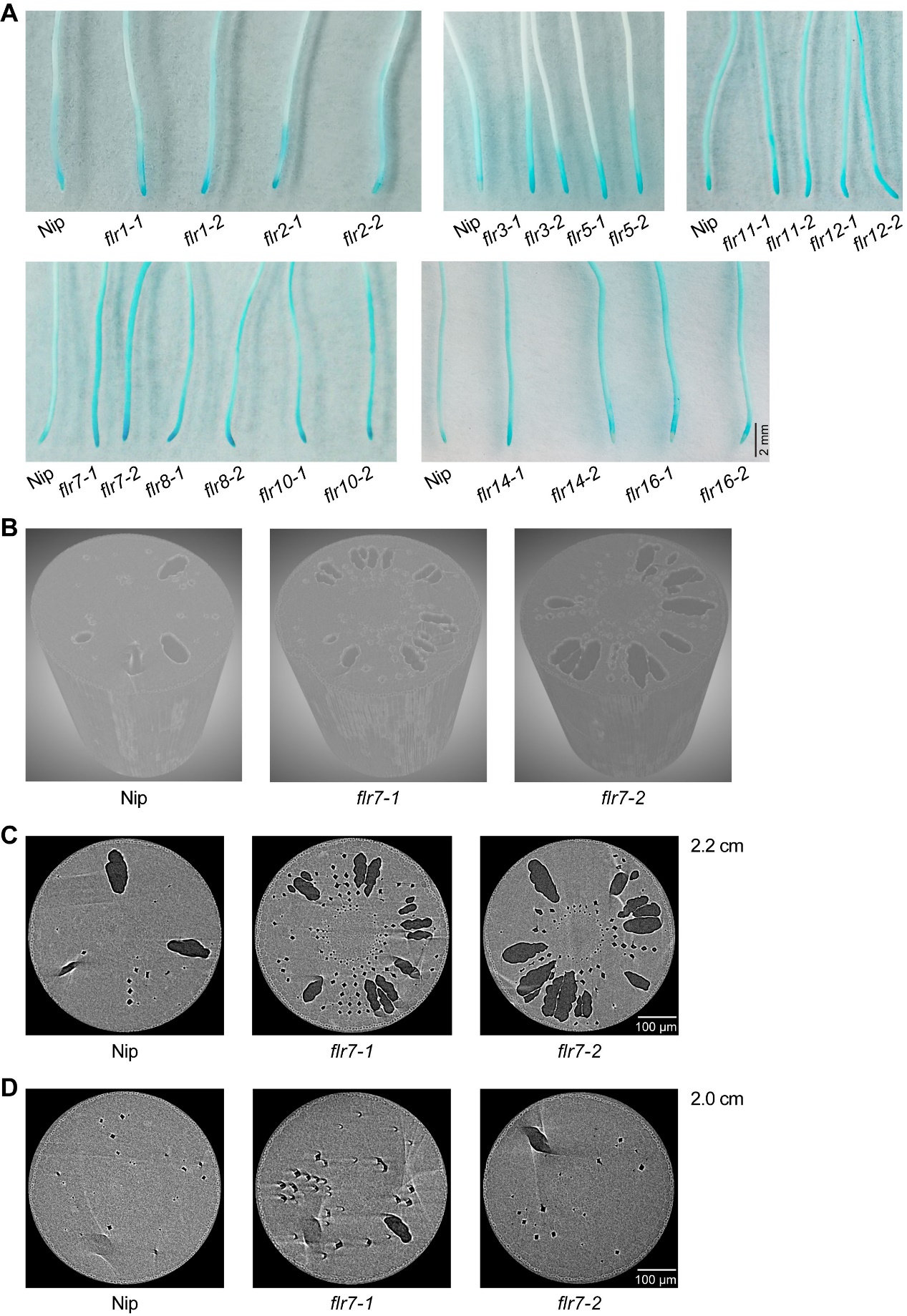


**Fig. S1 Representative images of root methylene blue staining and aerenchyma formation by three-dimensional X-ray microscopy. A,** Representative images of methylene blue staining of radial oxygen loss (ROL) from the roots of Nip and mutants. Blue indicates oxygen. **B,** Original 3D images in **Fig. 1E**. **C** **and D,** Root cross-sections at 2.2 cm (**C**) and 2.0 cm (**D**) away from the root tip.


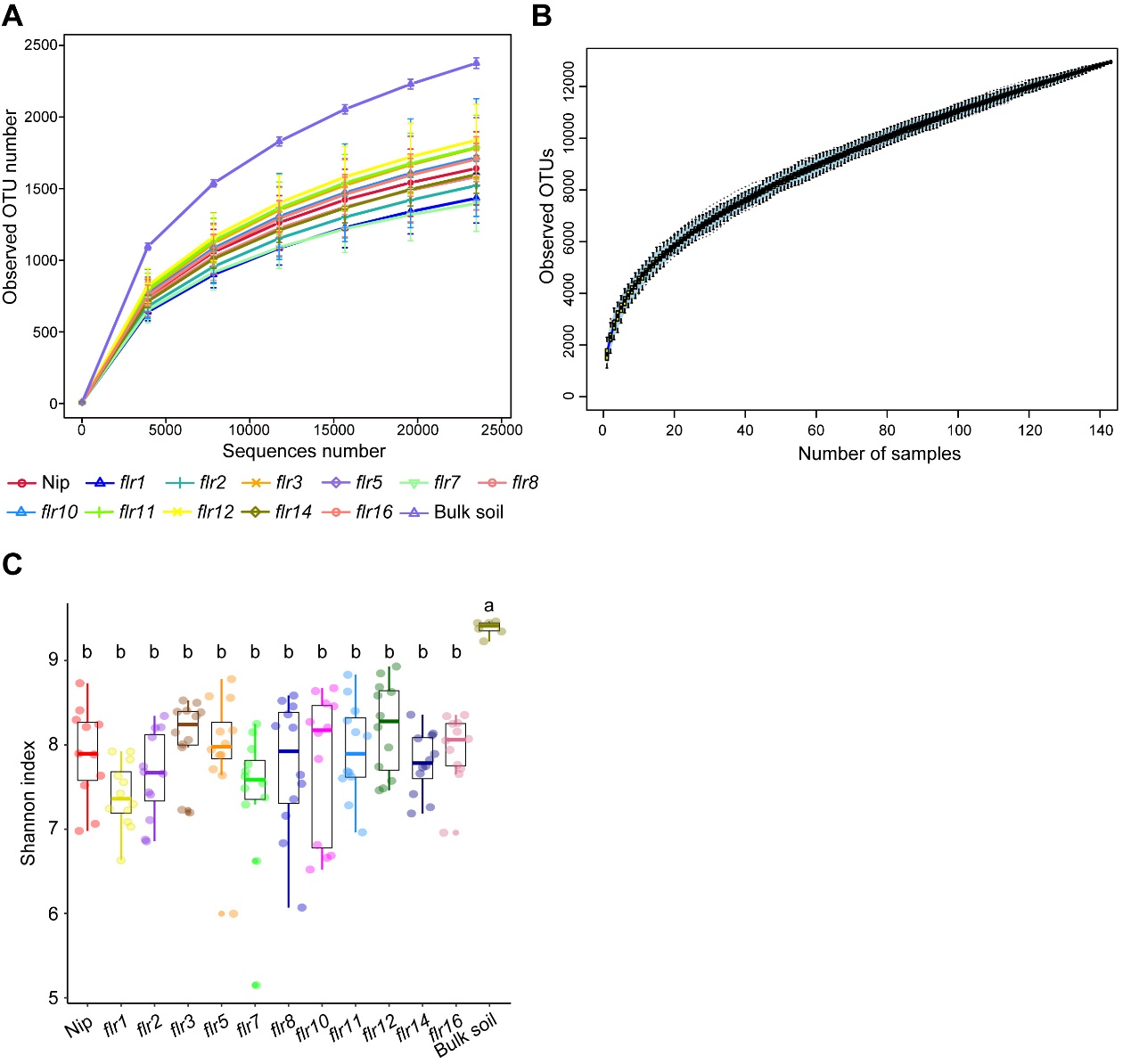


**Fig. S2 Coverage of members in the root bacterial microbiota. A,** The rarefaction curves of detected bacterial OTUs of the root microbiota from Nipponbare (Nip), 11 FLR mutants, and bulk soil approach the saturation stage with increasing sequencing depth. Each vertical bar represents the standard error. **B,** Species accumulation boxplot of detected bacterial species of the root microbiota approaching the saturation stage with increasing numbers of samples, indicating that the number of samples for root microbiome analysis was adequate. **C,** Shannon index of the root microbiota from Nip, 11 FLR mutants, and bulk soil. The horizontal bars within boxes represent medians. The tops and bottoms of the boxes represent the 75th and 25th percentiles, respectively. The upper and lower whiskers extend to data no more than 1.5× the interquartile range from the upper edge and lower edge of the box, respectively. Data were analysed by ANOVA with Tukey’s HSD test and exact *p* values are provided in Table S6. The numbers of replicated samples in this figure are as follows: Nip (n = 11), each FLR mutant (n = 12), and bulk soil (n = 6).


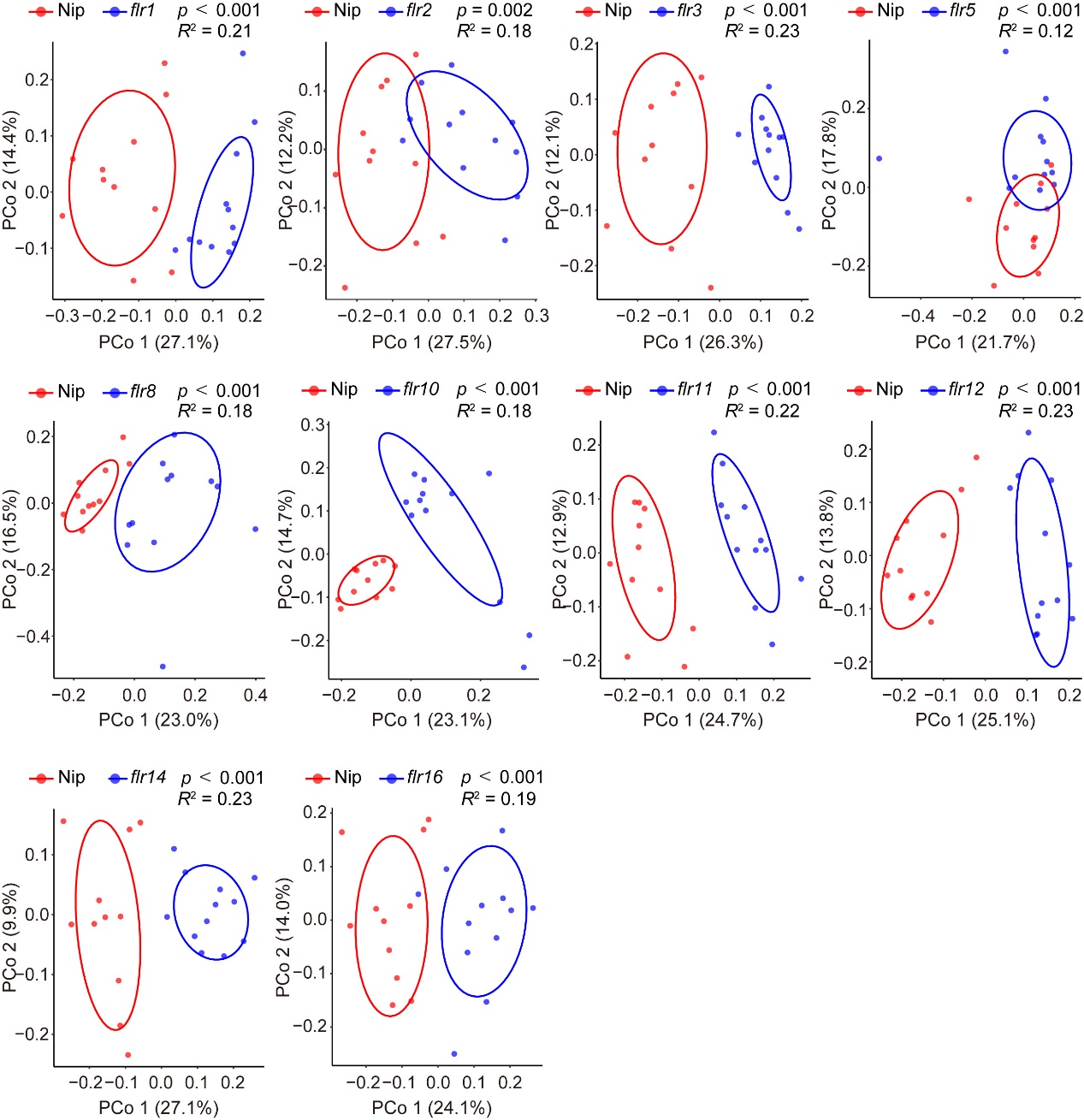


**Fig. S3 Unconstrained PCoA with Bray–Curtis dissimilarity between FLR mutants and Nip.** PERMANOVA was performed by Adonis, and ellipses covered 68% of the data for each rice genotype.


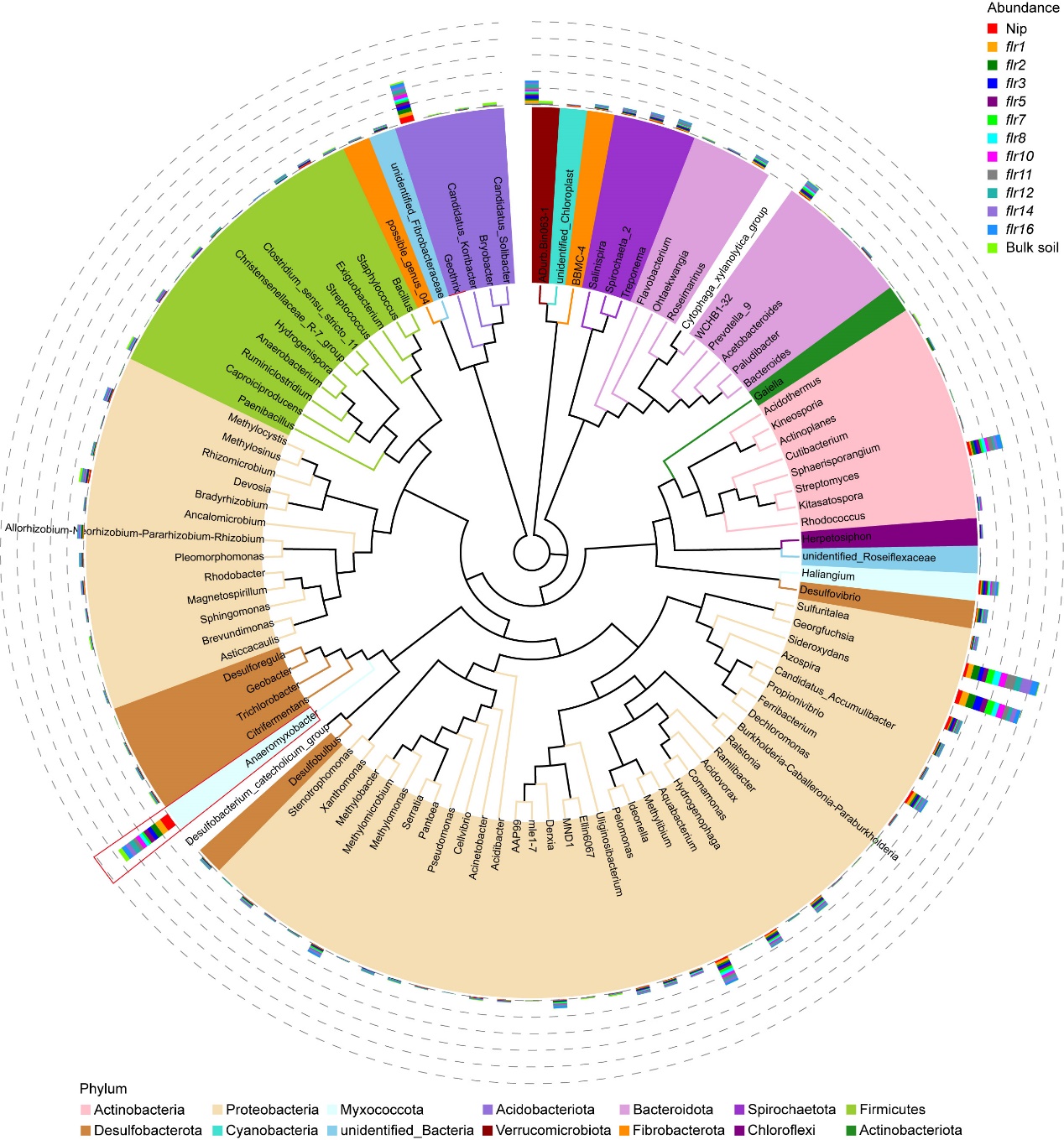


**Fig. S4 Phylogenetic relationships of the root microbiota of Nip and FLR mutants.** Phylogenetic relationship and abundance distribution of the top 100 genera in terms of relative abundance. The colors of the branches and sectors indicate their corresponding phyla, and the stacked bars outside the sector ring indicate the relative abundance of the genus in different samples. The most dominant genus is marked with red boxes.


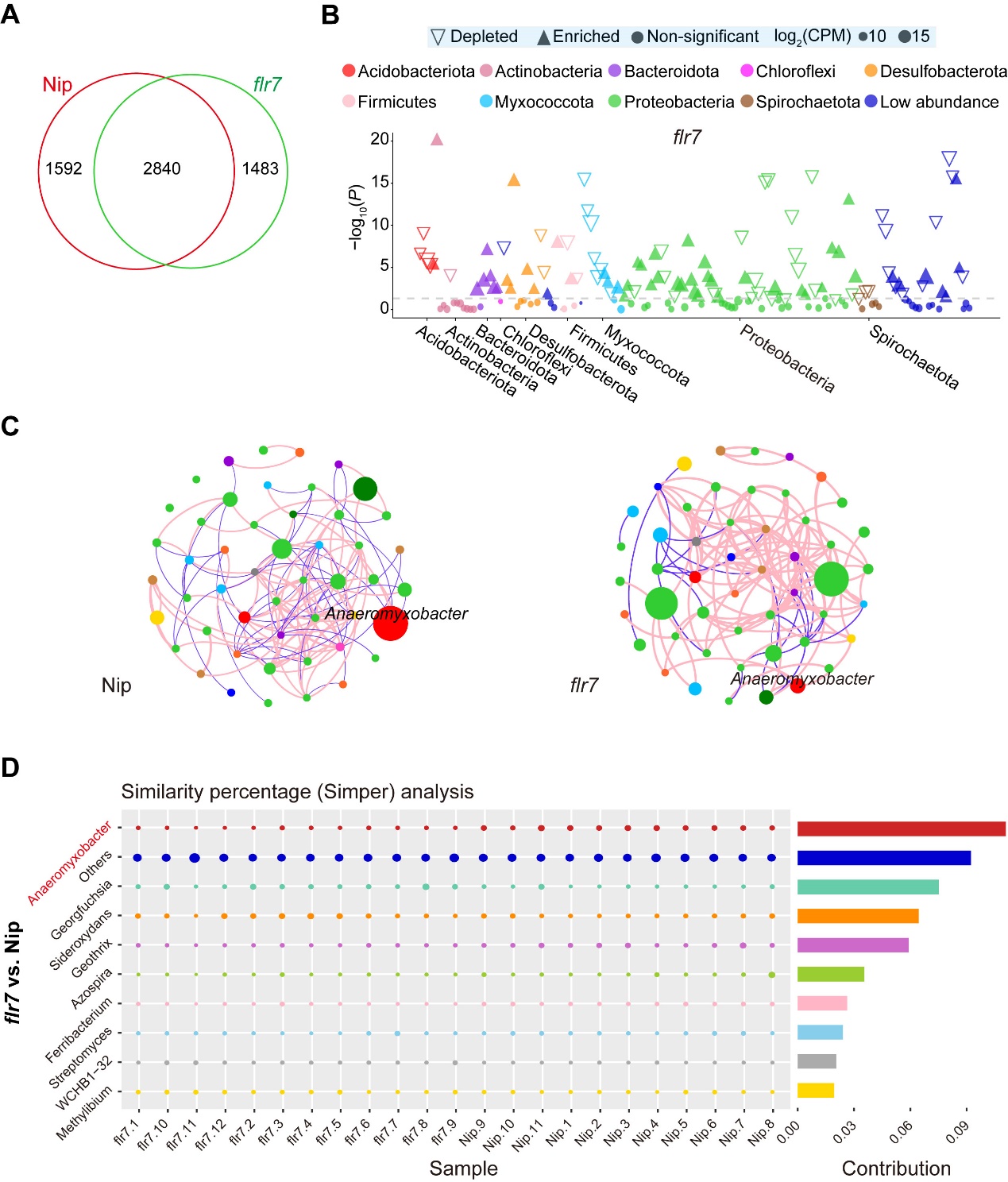


**Fig. S5 The root microbiota underwent dramatic changes in *flr7* compared to Nip. A,** Venn plot depicting the number of OTUs enriched and depleted in *flr7* compared to Nip. **B,** Manhattan plot showing OTUs enriched or depleted in *flr7* compared to Nip. Each dot or triangle represents a single OTU, and enriched or depleted OTUs are represented by filled or empty triangles, respectively (FDR adjusted *p* < 0.05, Wilcoxon rank sum test). OTUs are arranged in taxonomic order and colored according to phylum. CPM, counts per million. **C,** Co-occurrence networks of Nip and *flr7* root bacteria. Each node represents a genus, and the nodes are colored according to bacterial phylum. Node size indicates the relative abundance, and edge color represents positive (pink) and negative (blue) correlations. **D,** Simper analysis showing the contribution of genera to the difference in the root microbiota between Nip and *flr7*. The top 10 contribution rankings are presented. The bubble size represents the relative abundance of the genus in the corresponding sample.


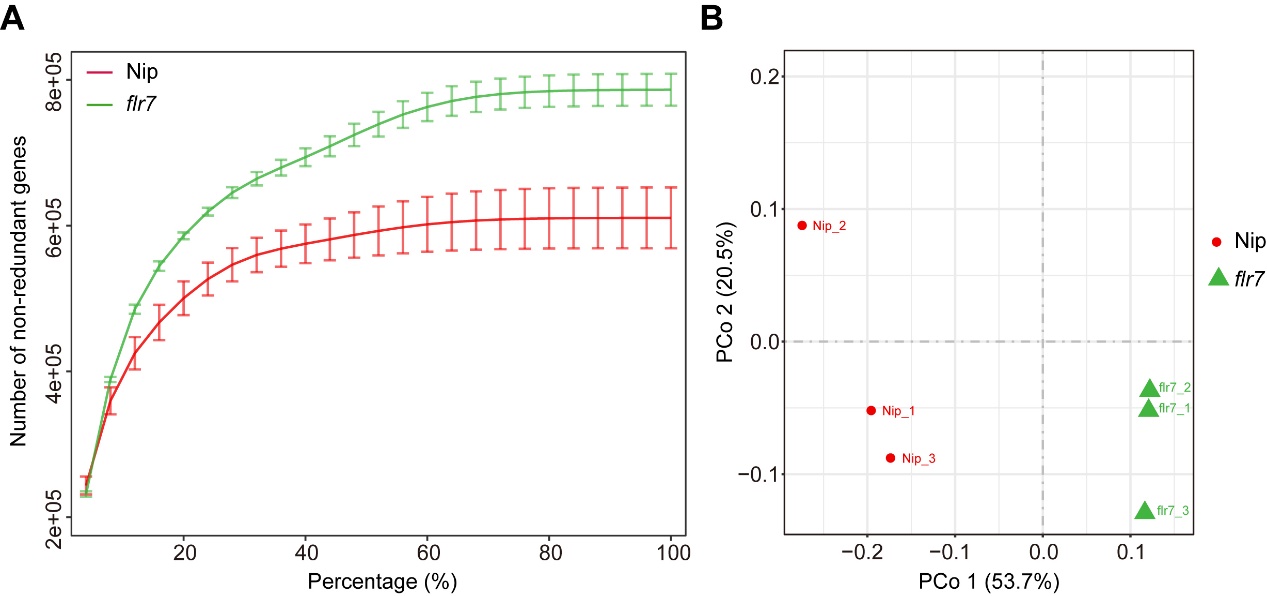


**Fig. S6 Gene coverage and species differences in metagenomic sequencing. A,** Rarefaction curves of nonredundant genes in the metagenomes reached the saturation stage with increasing sequencing depth, indicating that the sample sequencing depth for metagenomic analysis was adequate. **B,** Unconstrained PCoA with Bray–Curtis dissimilarity showing species differences between the Nip and *flr7* root microbiomes.

**
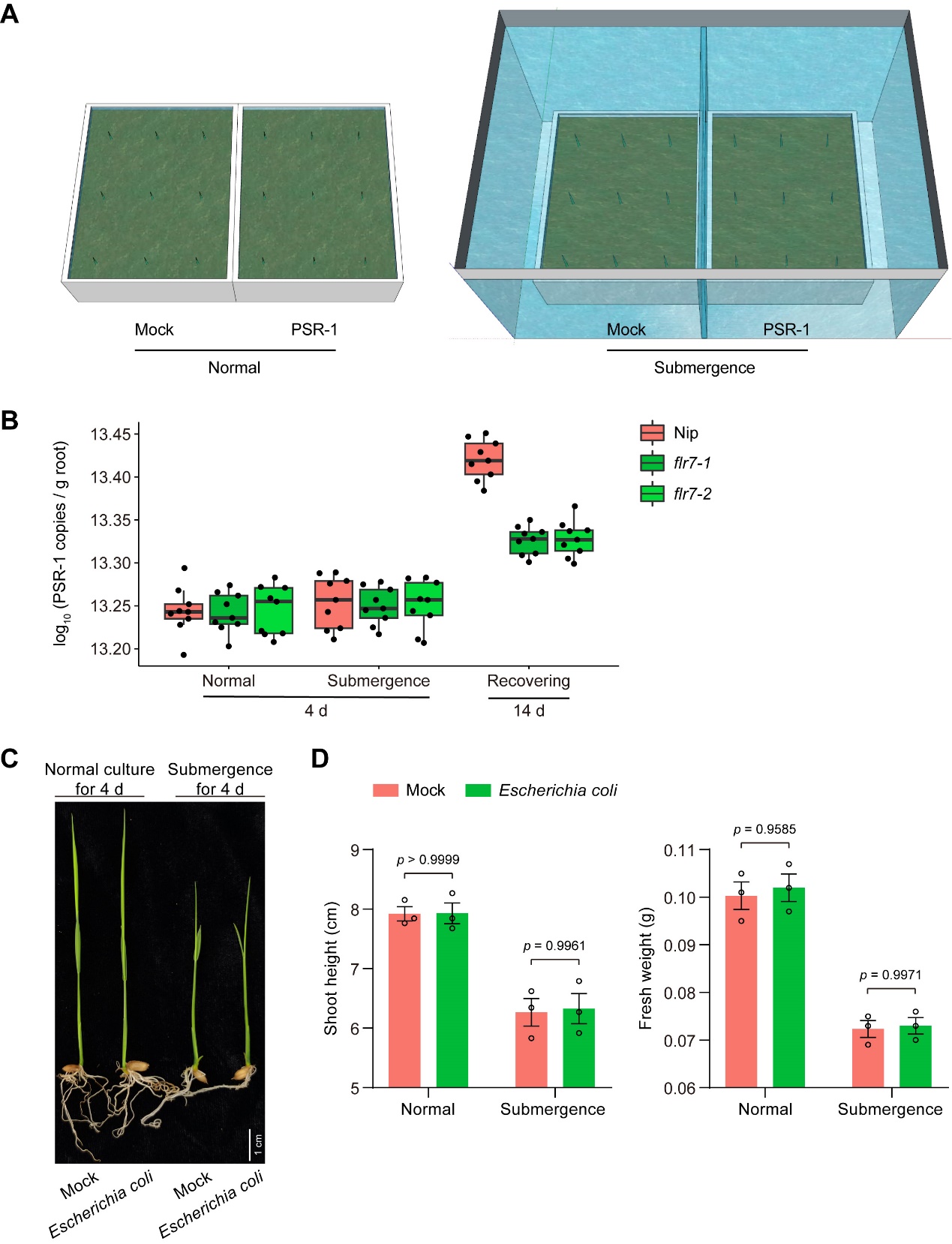
**

**Fig. S7 Diagram and control bacterial treatments for the submergence assay. A,** Diagram of normal culture and submergence treatment after rice root inoculation with *Anaeromyxobacter* strain PSR-1. **B,** Bacterial copies of *Anaeromyxobacter* sp. PSR1 in the rhizosphere of inoculated rice after normal culture, submergence treatment and recovery treatment. The numbers of replicated samples are as follows: Nip (n = 9), *flr7-1* (n = 9), and *flr7-2* (n = 9). Box plots show the median with upper and lower quartiles, and whiskers present the 1.5× interquartile range. **C,** Representative images of rice on the indicated days in normal culture and submergence treatment after root inoculation with the *Escherichia coli* strain TOP10. **D,** Shoot height and fresh weight of rice for each treatment in **C**. In **D**, data are the mean ± s.e.m. of three biological replicates. Statistical significance was determined by ANOVA with Tukey’s HSD test, and *p* values are indicated.


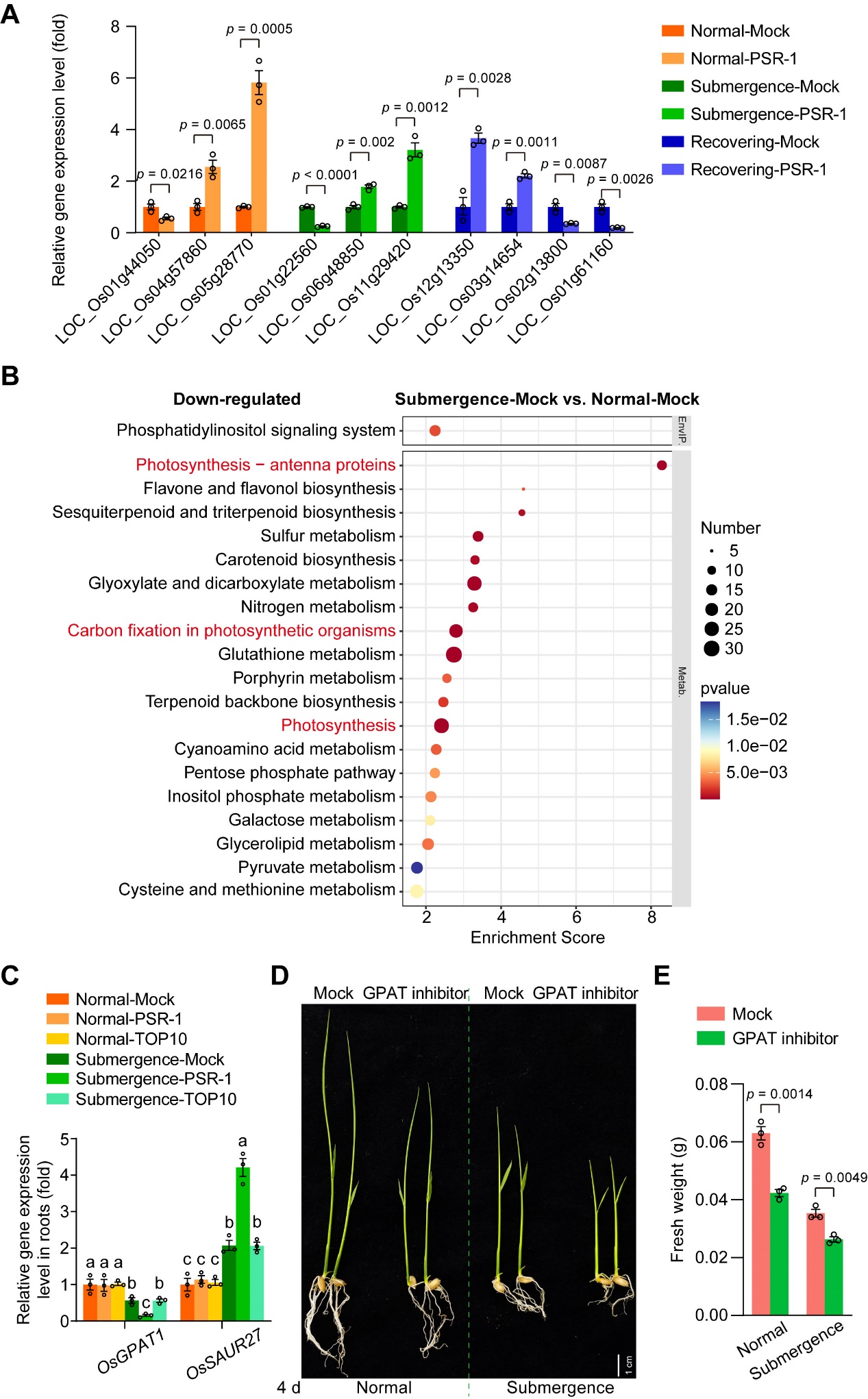


**Fig. S8 Validation and KEGG enrichment analyses of transcriptomes and effects of the GPAT inhibitor in rice. A,** The relative expression levels of 10 significantly differentially expressed genes obtained from transcriptome analysis were determined by qRT‒PCR. **B,** Top 20 pathways from KEGG enrichment analysis based on the *p* values for genes that showed downregulation with the submergence-mock treatment compared with the normal-mock treatment. **C,** Relative expression levels of *OsGPAT1* and *OsSAUR27* in rice roots. **D,** Representative images of rice treated with 50 μM GPAT inhibitor FSG67 in normal culture and submergence treatment conditions. DMSO (0.5%; v/v; mock) was used as a negative control. **E,** Fresh weight of rice for each treatment in **D**. In **A**, **C** and **E**, data are presented as the mean ± s.e.m. of three biological replicates. In **A** and **E**, data were analysed by Student’s *t-*test, and *p* values are indicated. In **C**, different letters above the bars indicate significant differences (*p* < 0.05) determined by ANOVA with Tukey’s HSD test. Exact *p* values are provided in Table S6.
