# Supplementary material for: Rice receptor kinase FLR7 regulates rhizosphere oxygen levels and enriches the dominant Anaeromyxobacter that improves submergence tolerance in rice
Source: ISME J. 2024 Jan 23;18(1):wrae006. doi: 10.1093/ismejo/wrae006 (PMC10900889; doi:10.1093/ismejo/wrae006)
Supplement: Supporting_information_for_materials_and_methods_wrae006 [file supporting_information_for_materials_and_methods_wrae006.docx]

**Supporting information for materials and methods**

**Analysis of the expression of hypoxia-inducible marker genes in roots**

Total RNA from the roots of *flr7* and Nip grown in soil for 20 days was extracted using TRIzol reagent (TaKaRa, Japan). qRT‒PCR was performed using the CFX96 Touch Real-Time PCR Detection System with SYBR Premix ExTaq II. *OsActin1* was used as a reference, and relative expression levels (fold changes) were determined with the 2^−ΔΔCt^ method. The primers used for *OsActin1*, *OsPCO1*, *OsPDC1*, *OsLBD41*, and *OsADH* are listed in **Table S2**.

**Metagenomic analysis**

DNA from three Nip root samples and three *flr7* mutant samples was sequenced on the NovaSeq 6000 platform using a paired-end 150 base pair (bp) sequencing strategy, with an average of 16.43 Gb per sample. All the raw data were trimmed by Trimmomatic v.0.36 [54], and then the clean reads were aligned against the *O. sativa* genome (gi: 996703420; accession: NW_015379174.1) to remove host contamination using Bowtie2 v.2.2.9 [55]. The remaining reads (with an average of 9.28 Gb per sample) were assembled by MEGAHIT v.1.1.2 [56]. Assembled contigs with lengths less than 500 bp were discarded in the following analysis. Filtered contigs were aligned against the *O. sativa* genome to remove host contamination with blastn-2.2.31. Gene prediction for contigs was performed using Prodigal v.2.6.3 [57], and nonredundant gene sets were built using Cd-hit v.4.6.7 with 95% identity and 90% coverage [58]. Clean reads of each sample were aligned against the nonredundant gene set using Bowtie2 with 95% identity, and the relative abundance of genes was calculated. The nonredundant genes were annotated with the NR, KEGG, and GO databases with an e-value of 1e-5. The taxonomy of the species was obtained according to the NR library, and the relative abundance of the species was calculated using the corresponding abundance of the genes. The relative abundance of functions from KEGG or GO analyses was generated by summing the gene abundances. The PCoA of the species abundance spectrum was carried out using the vegan, ape, and ggplot2 packages in R.

**Determination of *Anaeromyxobacter* abundance in the rice rhizosphere**

The 16S rRNA gene of *Anaeromyxobacter* sp. PSR-1 was amplified from the genomic DNA using the universal primers 27F and 1492R and cloned and inserted into the pRSF-Duet vector. The copy number of this plasmid was calculated according to the concentration measured by Nanodrop. A 10-fold dilution series of the plasmid was used as a template for qPCR to construct a standard curve between 16S rRNA gene copies and the Cq value. qPCR was performed using the CFX96 Touch Real-Time PCR Detection System (Bio-Rad, USA) with SYBR Premix ExTaq II (Takara, Japan). The qPCR primers used for the PSR1 16S rRNA gene are listed in **Table S2**.

*Anaeromyxobacter* sp. PSR-1 was added to sterilized Kimura B nutrient solution and mixed with autoclaved sand in sealable transparent boxes. Ten-day-old sterile rice mutants and Nip seedlings with uniform growth on MS agar medium were transplanted into the sand in the boxes with a depth of 0.5, 2.5, or 4.5 cm of sterile water. Different depths of water were set to produce differential rhizosphere oxygen levels indicated by the oxygen indicator (0.5 cm: 0.35-0.45%, 2.5 cm: 0.2-0.3%, 4.5 cm: < 0.1%; Mitsubishi, Japan). Each genotype in each treatment contained nine replicates. Plants were grown under long-day conditions (16 h light/8 h dark cycles) at 26 °C. After 30 days, the rice roots were removed, washed and weighed. Root DNA was extracted with hexadecyltrimethylammonium bromide (Sigma, USA) and used for qPCR to quantify the 16S rRNA gene copies of PSR1 according to the standard curve.

**Root inoculation with *Anaeromyxobacter* and submergence assay**

*Anaeromyxobacter* sp. PSR-1 was prepared as a suspension of OD_600_ = 0.4 using sterile water, in which roots of 4-day-old sterile Nip and *flr7* were submerged for one hour. Rice seedlings with uniform growth whose roots were submerged in sterile water were used as negative controls (mock). The seedlings were then transplanted into autoclaved sand mixed with Kimura B nutrient solution in boxes 15 cm long, 9 cm wide and 6.5 cm high, and 500 μL of bacterial suspension was applied to each root inoculated with PSR-1. Normal culture was set to maintain a depth of 2.5 cm of sterile water in the boxes, which provided a hypoxic environment for PSR-1. For the submergence treatment, the boxes were placed into large transparent boxes 32 cm long, 24 cm wide and 19 cm high filled with sterile water **(Fig. S7A)**. All of these rice seedlings were grown in a 75% alcohol-treated incubator at 26 °C. After 4 days of culture, the submergence treatment was allowed to recover to normal culture conditions. The shoot height and fresh weight of rice and the colonization of PSR-1 after normal culture, submergence treatment and recovery treatment were measured. Each treatment contained nine plants. A facultative anaerobic control bacterium, *E. coli* TOP10 (OD_600_ = 0.4), was also used to inoculate Nip roots in the submergence assay to rule out the possibility that any microbes contributed.

**Transcriptome sequencing analysis**

PSR-1-inoculated or uninoculated Nip seedlings under normal culture for 4 days, submergence treatment for 4 days, and recovery treatment for 14 days were collected, and total RNA was extracted using a mirVana miRNA Isolation Kit (Ambion, AM1561). RNA integrity was assessed using an Agilent 2100 Bioanalyzer (Agilent Technologies, Santa Clara, CA, USA). Libraries were constructed and sequenced using a HiSeq X Ten (Illumina) by OE Biotech Co., Ltd. (Shanghai, China). Three replicates of each treatment were included in the analysis. The raw data were processed using Trimmomatic v0.36 [54]. Clean reads were obtained by removing low-quality reads and were mapped to the rice genome Os-Nipponbare-Reference-IRGSP-1.0 using HISAT2 v2.2.1.0 [59]. Bioinformatics analysis was performed according to Liu *et al*. [29]. Significantly differentially expressed genes (DEGs) were identified with the DESeq2. *p* < 0.05 and fold change > 1.5 or < 0.5 were set as the thresholds for significant differential expression. KEGG and GO enrichment analyses were performed using R based on the hypergeometric distribution. The relative expression levels of 10 DEGs were determined by qRT‒PCR to validate the transcriptomic data, and the relative expression levels of *OsGPAT1* and *OsSAUR27* in roots were also further determined by qRT‒PCR. The primers used for these genes are listed in **Table S2**.

**Analysis of the effect of a GPAT inhibitor on rice growth**

A total of 12.5 mL of autoclaved sand was mixed with 10 mL of sterile Kimura B nutrient solution containing 50 μM GPAT inhibitor FSG67 in a 50 mL Falcon tube. The inhibitor solvent DMSO (0.5%; v/v) was used as a negative control. Nip seedlings with uniform growth were transplanted into the tubes for normal culture or submergence treatment. For the submergence treatment, an additional 30 mL of sterile water containing 50 μM FSG67 was added to the tube. After 4 days of culture, rice shoot height and fresh weight were measured. Each treatment contained six plants.
